# Type 1 Kounis Syndrome in Patient with Idiopathic Anaphylaxis

**DOI:** 10.1155/2017/1089023

**Published:** 2017-01-31

**Authors:** Tajda Keber, Jana Makuc, Gregor Sekavčnik

**Affiliations:** Department of Internal Medicine, General Hospital Slovenj Gradec, Gosposvetska 1, SI-2380 Slovenj Gradec, Slovenia

## Abstract

Kounis syndrome represents the concurrence of acute coronary syndromes or anginal pain with allergic, hypersensitivity, and anaphylactic reactions. It can be associated with normal coronary angiogram or preexistent coronary pathology. Idiopathic anaphylaxis is defined as anaphylaxis without any identifiable precipitating agent or event. We present a case of male who experienced attacks of dyspnoea, hypoxemia, hypotension, purple-red skin, and chest pain over several years. He was diagnosed with idiopathic anaphylaxis. Based on the pattern of chest pain of ischemic origin during the attacks he was retrospectively diagnosed with Kounis syndrome.

## 1. Introduction

Chest pain and dyspnoea represent common complaints in emergency departments. Several most common life-threatening cardiovascular causes are usually searched for or ruled out in emergencies. Patients with rare or unusual aetiologies of complaints represent a minority and are often misdiagnosed and suboptimally treated. If the complaint has chronic nature patients are repeatedly sent to emergency departments. They are examined by different physicians with different ideas of underlying aetiology. The path to establish a right diagnosis is therefore long and can lead through many unnecessary diagnostic procedures. Kounis syndrome is not a rare aetiology based on literature but remains overlooked due to focus on the main complaint at the moment of presentation by the threating physician. Here we present a patient with combination of pathologies which can represent a dilemma for a threating physician in emergency situation.

## 2. Case Report

Overweight male smoker allergic to metamizol with known dyslipidaemia was examined due to dyspnoea with acute respiratory insufficiency multiple times over several years. The attacks started approximately at the age of forty-eight. He was not receiving any regular therapy at that time. He was tachycardic and hypotensive during attacks; his skin was coloured purple-red. His complaints were dyspnoea, nausea, and chest pain. He also claimed to have several weaker attacks at home approximately twice monthly.

In the course of one of the attacks he fulfilled scintigraphic diagnostic criteria for pulmonary embolism. He was receiving oral anticoagulant treatment for one year.

During one of the attacks he suffered acute myocardial infarction with ST-segment elevation (STEMI). He underwent coronarography which showed normal coronary arteries. It was concluded he suffered coronary artery spasm which caused STEMI. Therapy with acetylsalicylic acid and nondihydropyridine calcium channel blocker was started. Because of his complaints of occasional chest pain perfusion myocardial scintigraphy was performed. It showed no reversible myocardial ischemia and fully viable myocardium. He terminated calcium antagonist treatment.

Due to patients' physical appearance during the attack catecholamines' values in urine sample were scanned several times. They were always under the cut-off value. Based on several under-cut-off values of 5-HIAA and negative scintigraphic scan for somatostatin receptors we also ruled out carcinoid syndrome.

In the course of one of the attacks a serum tryptase sample was obtained. The tryptase was markedly elevated (57,5 *μ*g/L) in comparison to tryptase value during asymptomatic state which was normal (4 *μ*g/L). We therefore performed bone marrow biopsy which ruled out systemic mastocytosis.

In suspicion of allergic aetiology of unknown origin treatment with acetylsalicylic acid was terminated. He was diagnosed with idiopathic anaphylaxis after he underwent a work-up for common allergies. He was advised not to use nonsteroid anti-inflammatory drugs. Treatment with antihistaminic loratadine was started which he randomly terminated after few months. After the termination of antihistaminic treatment he experienced worse symptoms during the attacks again. During one of the attacks after diagnosis of IA was made he received intramuscular adrenalin injection. His chest pain was aggravated for several minutes.

He is currently on antihistaminic. Attacks in mild form are still present.

The diagnosis of Kounis syndrome was made retrospectively after studying the pattern of repeating stenocardic chest pain experienced during anaphylactic events.

## 3. Discussion

Kounis syndrome is defined as the concurrence of acute coronary syndromes or anginal pain in conditions associated with mast-cell and platelet activation involving interrelated and interacting inflammatory cells in the setting of allergic or hypersensitivity and anaphylactic or anaphylactoid insults [1]. Causes capable of inducing Kounis syndrome include numerous drugs, environmental exposures, and conditions such as asthma, idiopathic anaphylaxis (as in the case of our patient), and mastocytosis [2].

During hypersensitivity, degranulation of mast cells releases inflammatory mediators into the systemic circulation. Most of these mediators have important cardiovascular activity. Histamine induces coronary vasoconstriction and tissue factor expression and consequently activates platelets. Neutral proteases can activate matrix metalloproteinases, which can degrade the collagen cap and induce plaque erosion and rupture. Tryptase has thrombotic and fibrinolytic properties. Many mediators have vasoconstrictive properties and may worsen coronary vasospasm [1].

The diagnosis of Kounis syndrome is based on clinical symptoms and signs as well as on laboratory, electrocardiographic, echocardiographic, and angiographic evidence. Increased serum tryptase, histamine, cardiac enzymes, and cardiac troponins are particularly helpful findings. Measuring cardiac troponins in all patients admitted to the emergency department with acute allergic reactions in order to timely diagnose and appropriately manage a potential cardiac injury manifesting as Kounis syndrome has been already suggested. Echocardiography and coronary angiography are necessary in diagnosing cardiac wall abnormalities including takotsubo cardiomyopathy and delineating the coronary anatomy [3].

Our patient was retrogradely diagnosed with Kounis syndrome as the connection between allergic features and chest pain was established. Nevertheless, he experienced STEMI with normal coronary angiogram in the course of one of the attacks.

Three variants of Kounis syndrome have been described: type I variant includes normal or nearly normal coronary arteries without risk factors for coronary artery disease and with the acute release of inflammatory mediators that may induce either coronary artery spasm without increased cardiac enzymes and troponins or coronary artery spasm progressing to acute myocardial infarction with raised cardiac enzymes and troponins. Type II variant includes culprit but quiescent preexisting atheromatous disease in which the acute release of inflammatory mediators may induce either coronary artery spasm or coronary artery spasm together with plaque erosion or rupture manifesting as acute myocardial infarction. Type III variant includes coronary artery stent thrombosis [1].

Our patient is classified as type I variant.

True frequency of Kounis syndrome is unknown. However, during insect sting challenge study 9,5% of healthy volunteers developed chest pain with electrocardiographic abnormalities consistent with acute myocardial ischemia [4].

Idiopathic anaphylaxis (IA) is defined as anaphylaxis without any identifiable precipitating agent or event. The clinical manifestations include urticaria, angioedema, hypotension, tachycardia, wheezing, stridor, pruritus, nausea, vomiting, flushing, diarrhoea, dysphagia, light-headedness, and loss of consciousness. Patients usually tend to have the same manifestations on repeated episodes. IA is a diagnosis of exclusion. Approximately 40% of patients are atopic. Serum tryptase (or urine histamine or its metabolite) will be elevated acutely [5].

Our patient experienced typical symptoms during the attacks. Serum tryptase was also elevated.

IA is classified according to the symptoms as well as the frequency of attacks. Patients who experience six or more episodes in a year or two or more episodes in 2 months are classified as IA-frequent (IA-F) [5].

Patients with IA must carry and know when and how to self-administer epinephrine. All patients with IA should be advised to avoid taking drugs that might complicate treatment or worsen an event (*β*-adrenergic blockers, angiotensin converting enzyme inhibitors, angiotensin blockers, monoamine oxidase inhibitors, tricyclic antidepressants, and nonsteroidal anti-inflammatory drugs) [5, 6]. If a patient is already known to experience frequent attacks of idiopathic anaphylaxis, empiric therapy with prednisone and H1 antagonist should be tried out. If the patient is asymptomatic, then prednisone can be tapered every 2 to 4 weeks until it is determined that the patient has had no more episodes of anaphylaxis and prednisone is not required or more episodes occur. This empiric approach has proven very effective in reducing the frequency and severity of future reactions. For reasons that are not clear, the vast majority of patients with idiopathic anaphylaxis gradually improve. Episodes decline in frequency and remissions occur in many instances [6].

Our patient is retrospectively classified as IA-F type. He had several attacks which vary in intensity approximately twice monthly. He was receiving antihistaminic for several months. After termination of antihistaminic, symptoms worsen. Despite lack of specific treatment with prednisone his attacks decline in frequency.

Treatment of Kounis syndrome is challenging because it needs to address both cardiac and allergic symptoms simultaneously and the drugs administered may worsen the allergy and aggravate heart function. In patients with type I variant, treatment of the allergic event alone may abolish symptoms. The use of hydrocortisone and H1 and H2 antihistamines is adequate. The administration of vasodilators such as calcium channel blockers may abolish hypersensitivity-induced vasospasm and they may be considered as the initial anti-ischemic agent [1, 7].

The potential effect of several drugs is debatable. Aspirin may aggravate an ongoing allergic reaction. Nitroglycerine may cause hypotension and tachycardia, which may further complicate allergic reactions, but seems safe if blood pressure is satisfactory. Epinephrine, which is the drug of choice and may save lives in anaphylaxis, may aggravate ischemia and worsen coronary vasospasm in Kounis syndrome. It is advised that sulphite-free epinephrine is given intramuscularly in aqueous solution when needed [8].

Our patient experienced worse stenocardia after intramuscular adrenalin injection which further consolidates the diagnosis.

Beta-blockers may induce more vasospasm due to unopposed *α*-adrenergic effect and may offset some of the beneficial effects of epinephrine. Opioids such as morphine, codeine, and meperidine given to relieve acute chest pain should be administered with extreme caution in patients with Kounis syndrome because they may induce massive mast-cell degranulation and aggravate allergic reaction. Paracetamol is not recommended, especially by intravenous administration, because it might cause severe hypotension due to a reduction in cardiac output. Fentanyl and its derivatives are weak mast-cell triggers and may be considered as the drug of choice when narcotic analgesia is necessary [1, 7].

Our patient represents a special challenge due to idiopathic nature of anaphylactic reactions and frequent chest pain attacks. Optimal treatment for combination of his pathologies is still debatable.

## References

[1] Kounis N. G. (2013). Coronary hypersensitivity disorder: the kounis syndrome. *Clinical Therapeutics*.

[2] Kounis N. G., Hahalis G., Manola A., Kourelis T., Theoharides T. C. (2008). Kounis syndrome (allergic angina and allergic myocardial infarction). *Angina Pectoris: Etiology, Pathogenesis and Treatment*.

[3] Kounis N. G. (2016). Natural paradigm: kounis hypersensitivity associated acute coronary syndrome. *Achaiki Iatriki*.

[4] Lopez P. R., Peiris A. N. (2010). Kounis syndrome. *Southern Medical Journal*.

[5] Blatman K. H., Ditto A. M. (2012). Chapter 25: idiopathic anaphylaxis. *Allergy and Asthma Proceedings*.

[6] Greenberger P. A., Lieberman P. (2014). Idiopathic anaphylaxis. *The Journal of Allergy and Clinical Immunology: In Practice*.

[7] Rodrigues M. C. A. L., Coelho D., Granja C. (2013). Drugs that may provoke Kounis syndrome. *Brazilian Journal of Anesthesiology*.

[8] Kounis N. G. (2016). Kounis syndrome: an update on epidemiology, pathogenesis, diagnosis and therapeutic management. *Clinical Chemistry and Laboratory Medicine*.

